# Microultrasound imaging for accuracy of diagnosis in prostate cancer: a single‑center observational study

**DOI:** 10.20452/wiitm.2025.17978

**Published:** 2025-09-08

**Authors:** Michael Grynkiewicz, Nicolò Maria Buffi, Vittorio Fasulo, Fabio De Carne, Edoardo Beatrici, Nicola Frego, Maciej Wiewiora, Giovanni Lughezzani

**Affiliations:** Department of Urology, Pediatric Urology, and Robot Assisted Minimally Invasive Urology Sozialstiftung Bamberg Hospital Bamberg, Bamberg Germany; Department of Urology, IRCC Humanitas Research Hospital, Milano, Italy; Department of General Surgery, Vascular Surgery, Angiology, and Phlebology, Faculty of Medical Sciences in Katowice Medical University of Silesia Katowice Poland

**Keywords:** microultrasound imaging, prostate cancer, targeted biopsy

## Abstract

**INTRODUCTION:**

Research results suggest that high-resolution ultrasonography (microUS) can be used to estimate the risk of prostate cancer (PCa) and identify suspicious areas, enabling precise biopsy during examination.

**AIM:**

The aim of the study was to assess the usefulness of microUS in a population of patients with suspected PCa.

**MATERIALS AND METHODS:**

An observational single-center study on the application of microUS was conducted in a group of 439 patients. All examinations were performed using the ExactVu microUS system.

**RESULT:**

A total of 439 patients with suspected PCa underwent microUS examination. Following the examination, suspicious lesions (positive result) were identified in 196 patients (44.6%). Among them, 86 (43.8%) underwent multiparametric magnetic resonance imaging (mpMRI), and suspicious lesions were found in 36% of the cases. There was no major difference in the frequency of positive results on microUS and mpMRI (*P* <⁠0.4). Concordant results of mpMRI and microUS were observed in 71% of the patients. The accordance between microUS and mpMRI was more frequent than a lack of accordance (*P* <⁠0.01). There was no difference in the frequency of PCa detection between the 2 groups (*P* <⁠0.6). There was no difference in the frequency of PCa detection in the group with concordant microUS and mpMRI findings, as compared to the discordant group of patients (*P* <⁠0.6). In the group of 243 individuals with negative microUS results, 87 (35.8%) underwent mpMRI. Result concordance between negative mpMRI and microUS was 79%.

**CONCLUSION:**

A positive result from microUS, even when MRI is negative, may indicate a need for prostate biopsy. On the other hand, a negative result from microUS suggests a low likelihood of clinically significant PCa and thus a need for biopsy.

## INTRODUCTION

Prostate cancer (PCa) is one of the most common malignant tumors in men and one of the leading causes of cancer-related deaths worldwide.^1^ Diagnosing PCa at advanced stages of the disease is a major factor contributing to increased mortality.^2^ Early detection of PCa is essential to improve prognosis and reduce mortality. Multiparametric magnetic resonance imaging (mpMRI), considered the gold standard, is a key tool for assessing the risk of clinically significant PCa based on the standardized Prostate Imaging Reporting and Data System (PI-RADS) scale.^3^ Prostate biopsy under transrectal ultrasound (TRUS) or MR guidance is the basis for diagnosing PCa.

TRUS using conventional 5–10 MHz frequency transducers has been one of the most commonly used imaging techniques for both visualizing and performing prostate biopsies. Unfortunately, the rate of false-negative results is high, estimated at around 42%,^4^ which reduces the sensitivity and specificity of TRUS in detecting PCa, ranging from 50% to 92% and from 46% to 91%,^5^ respectively. High-resolution ultrasonography (microUS) allows for high-quality imaging through the use of a probe with an ultrasound frequency of up to 29 MHz, enabling a more accurate assessment of prostate structure than conventional TRUS. Both the specificity and sensitivity of the prostate lesion assessment are significantly higher with the microUS technique than conventional TRUS.^6^

In the first study comparing conventional TRUS with microUS in patients with histopathologically confirmed PCa, higher sensitivity and specificity were found for microUS (65.2% vs 37.7% and 71.6% vs 65.2%, respectively).^7^ A systematic review of clinical studies^8^ found the sensitivity and specificity of microUS in prostate tumor diagnosis to be 91% and 49%, respectively. Another study reported that prostate biopsy under microUS guidance increased PCa detection by 28% in comparison with conventional TRUS.^9^ A recent review showed that the detection rates for clinically significant PCa with microUS are comparable to those of mpMRI-guided biopsies.^10^

The authors of the analysis suggest that current literature indicates microUS should replace conventional TRUS in prostate imaging and biopsy. However, it remains unclear whether microUS should be used alone or in combination with mpMRI to improve PCa detection.

## AIM

The aim of the study was to assess the usefulness of microUS in diagnosing PCa based on the correlation between positive microUS results and confirmed PCa diagnosis, and to evaluate the percentage of patients suspected of PCa who did not require further diagnostics based on a negative microUS result.

## MATERIALS AND METHODS

This observational study focused on the use of microUS in a group of 439 patients with suspected PCa. The study included patients with suspected PCa based on a positive digital rectal examination (DRE) and / or prostate specific antigen (PSA) and / or a positive mpMRI result, as well as patients enrolled under an active surveillance (AS) protocol. We included consecutive patients who met the inclusion criteria, which encompassed elevated serum level of total PSA (tPSA; cutoff value >4 ng/ml), abnormal findings on DRE, a relevant clinical history, with particular attention to whether the patient was already enrolled in the AS protocol, and positive findings on mpMRI, defined as PI-RADS score of 3 or higher. All the parameters were systematically assessed during the initial patient evaluation. Based on these criteria, eligible patients were subsequently offered further diagnostic assessment with microUS. Exclusion criteria were limited to situations in which the procedure could not be tolerated, primarily due to patient discomfort or a lack of patient consent to participate. The study was carried out between November 2018 and January 2023 in the Urology Department of Humanitas Research Hospital in Milan, Italy. The research was carried out in accordance with the ethical standards and the precepts outlined in the Declaration of Helsinki. The research protocol for the study was approved by the research ethics committee at IRCC Humanitas Research Hospital – Via Manzoni Rozzano (Milan; IRCC HRS -2871/18), and all participants provided their written informed consent.

All patients underwent microUS in an outpatient setting using the ExactVu microUS device (Exact Imaging, Markham, Canada). Prostate visualization was performed with the EV29L probe at a frequency of 29 MHz (Exact Imaging). The examination was done by an experienced urologist familiar with the prostate risk identification using micro-ultrasound (PRI-MUS) protocol, which stratifies the risk of PCa.^11^ The patients enrolled in the study were divided into 2 groups based on the transrectal microUS result: positive or negative, depending on the PRI-MUS score. A PRI-MUS score of 3 or higher was considered positive and suggestive of a potentially cancerous lesion. A score below 3 was classified as negative, indicating a very low risk of PCa. The concordance or discordance between microUS and mpMRI was also noted, depending on whether the lesions were detected or not in the same locations. The microUS results were collected in a standardized worksheet, which also included main clinical characteristics of each patient. All data were extracted and compiled into a database using an Excel spreadsheet, from which the following parameters were retrieved: prior biopsies, DRE results, PSA level, prostate volume, mpMRI and microUS results, concordance between microUS and mpMRI findings, and histopathological results of prostate biopsy. Subsequently, the clinical data of the patients were uploaded into an online clinical documentation platform wHospital (wHospital, Humanitas Research Hospital, Rozzano, Italy), used by the research center.

Biopsies were performed transrectally in the lithotomy position, as 1-day surgery, by 2 experienced urologists. Prior to the procedure, bowel preparation was advised and antibiotic prophylaxis was administered. Each patient received levofloxacin at a dose of 500 mg. The procedure was performed under local anesthesia using 2% lidocaine administered under ultrasound guidance. For patients with no prior biopsy, a 12-core biopsy scheme was used, with an additional targeted core based on MRI or microUS findings. For patients with a history of prior biopsy, only targeted biopsy was performed, with or without additional sextant biopsy. The specimens were analyzed by 2 specialized pathologists.

mpMRI (3T or 1.5T) was performed using an endorectal coil with at least 3 sequences as defined by PI-RADS: T2-weighted, dynamic contrast-enhanced, and diffusion-weighted image. The mpMRI images were evaluated and classified according to the PI-RADS version 2 protocol, considering each suspicious lesion with a PI-RADS score of 3 or higher.^12^

### Statistical analysis

Continuous variables are presented as median with maximum and minimum values. The χ^2^ test or Fisher exact test was performed to compare differences in the accordance between microUS and mpMRI. The κ coefficient was calculated to assess the agreement between microUS and mpMRI. Statistical analysis was conducted using SPSS software (SPSS 22.0, IBM, Armonk, New York, United States).

## RESULTS

A total of 439 patients aged between 59 and 72 years with suspected PCa underwent microUS examination. Median tPSA level and prostate volume were 5.73 ng/ml (range, 3.99–7.9 ng/ml) and 44 cm³ (range, 31–62.1 cm³), respectively.

Following the microUS examination, suspicious lesions (positive result) were identified in 196 patients (44.6%). Among them, 30 individuals (15.3%) were classified as PRI-MUS 3, 124 (63.2%) as PRI-MUS 4, and 43 (21.9%) as PRI-MUS 5. A total of 173 patients underwent mpMRI, with 65 (37.5%) having a positive result. Of those, 37 (59.7%) had the PI-RADS score 3, 15 (24.2%) PI-RADS 4, and only 5 (7.7%) had the PI-RADS score of 5. In 8 patients (12.3%), mpMRI result was positive but the PI-RADS score was not precisely specified.

**Figure 1:**
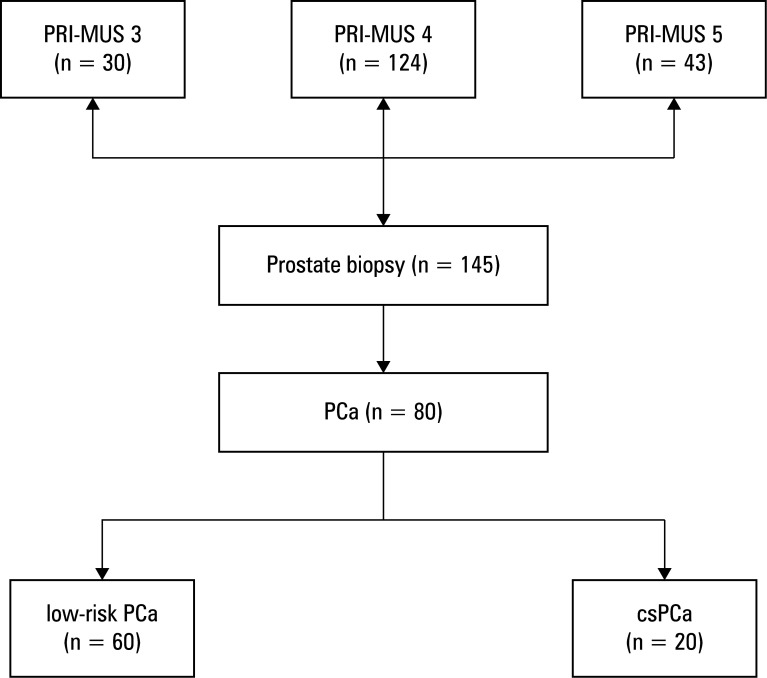
Results of prostate biopsy in the patients with a positive result of high-resolution ultrasound examination

Abbreviations: cs, clinically significant; PCa, prostate cancer; PRI-MUS, prostate risk identification using microultrasound

As many as 145 patients underwent biopsy based on targeted microUS findings. PCa was detected in 80 of them (55.2%), with 60 patients (75%) diagnosed with low-risk PCa (Gleason score [GS] <⁠6) and 20 (25%) with clinically significant PCa (csPCa; GS >7; Figure 1).

### Patients with positive results of high-resolution ultrasonography

**Figure 2:**
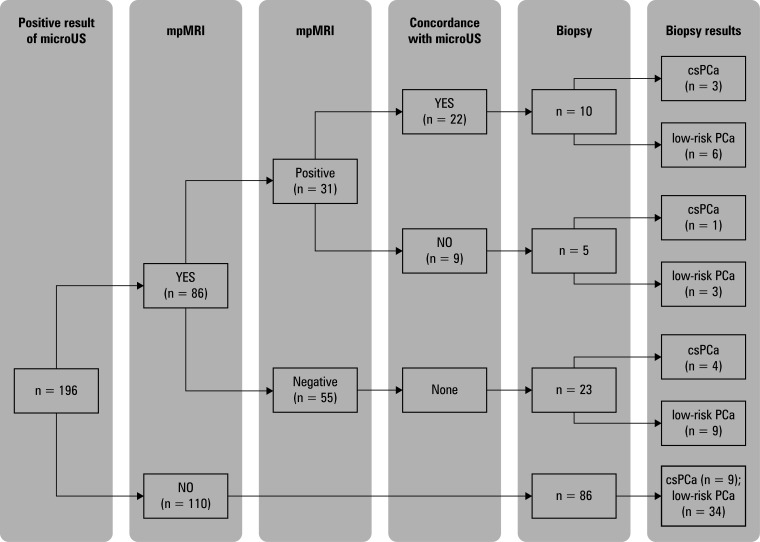
Schematic description of the diagnostic workup in the patients with a positive result of high-resolution ultrasound examination

Abbreviations: microUS, high-resolution ultrasonography; mpMPRI, multiparametric magnetic resonance imaging; others, see Figure 1

**Figure 3:**
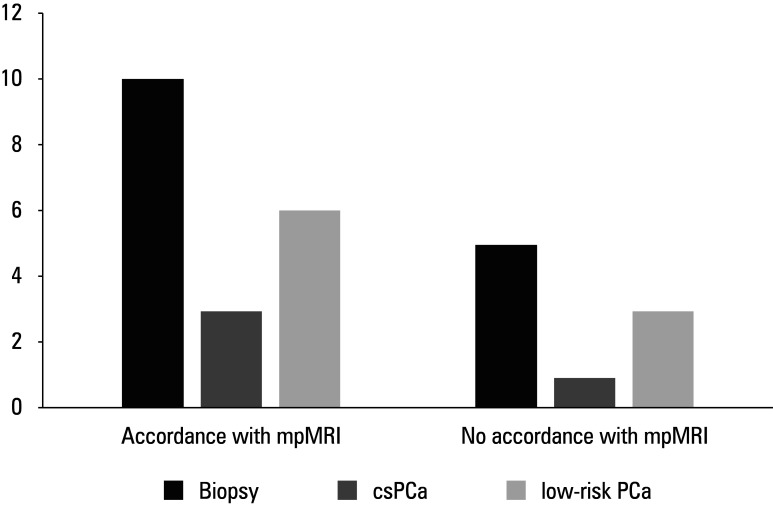
Biopsy results in the group of patients with positive results of high-resolution ultrasound and multiparametric magnetic resonance imaging

Abbreviations: see Figure 1 and Figure 2

In 196 patients (44.6%), microUS raised a suspicion of PCa. Among them, 86 individuals also underwent mpMRI, and suspicious lesions (PI-RADS ≥3) were found in 31 cases (36%). There was no major difference in the frequency of positive results from microUS and mpMRI (*P* <⁠0.5). The accordance between microUS and mpMRI findings was more frequent than a lack of accordance (*P* <⁠0.01), with the κ coefficient of 0.69. Concordant results of mpMRI and microUS were observed in 22 patients (71%). Biopsies were performed in 10 of them (45%), with PCa diagnosed in 9 cases (90%): 3 (33.3%) of csPCa and 6 (66.6%) of low-risk PCa. In 9 of the 31 patients with PI-RADS score 3 or higher (29%), mpMRI and microUS results were discordant. Among these, 5 (55.6%) underwent biopsy that showed csPCa in 1 person (20%) and low-risk PCa in 3 cases (60%; Figure 2). There was no difference in the frequency of PCa detection between the 2 groups (*P* <⁠0.6; Figure 3).

Of the 55 patients (64%) with negative mpMRI results, 23 (41.8%) underwent biopsy. In this subgroup, 13 (56.5%) were found to have PCa: 4 csPCa (30.8%) and 9 low-risk PCa (69.2%; Figure 2).

Among the 110 patients (56.1%) with positive microUS result who did not undergo mpMRI, 86 (78.2%) had targeted prostate biopsies based on microUS findings. PCa was confirmed in 43 cases (50%), including 9 cases (20.9%) of csPCa Figure 2.

### Patients with negative results of high-resolution ultrasonography

**Figure 4:**
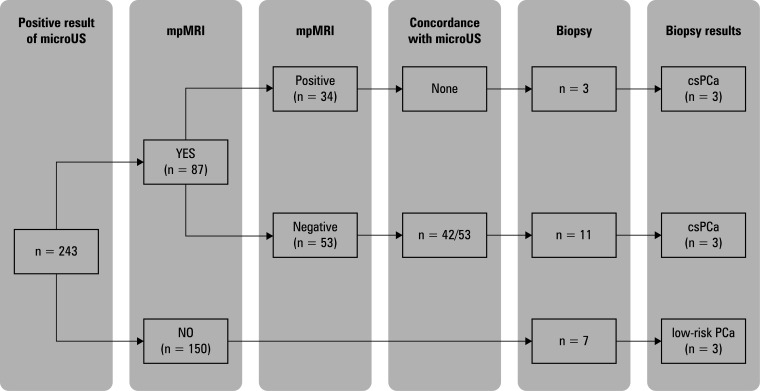
Schematic description of the diagnostic workup in the patients with a negative result of high-resolution ultrasound examination

Abbreviations: see Figure 1 and Figure 2

In the group of 243 patients with negative microUS results, 87 (35.8%) underwent mpMRI, with positive findings in 34 cases (39.1%; Figure 4). Of those, 3 patients underwent biopsy, and csPCa was detected in 1 of them. On the other hand, 53 patients (61%) had negative mpMRI results, with concordant microUS findings in 42 cases (79%). Biopsies were performed in 11 of these patients (20.7%), identifying 2 cases of csPCa and 5 cases of non-csPCa. Among the 156 individuals (64.1%) who did not undergo mpMRI examination, 7 (4.48%) underwent randomized biopsy, revealing 3 cases of low-risk PCa. All results are shown in Figure 4.

## DISCUSSION

Currently, mpMRI is considered the gold standard in PCa diagnosis. It is an accurate diagnostic tool, especially when biopsy results from conventional TRUS are negative despite high clinical suspicion of PCa.^13^ In comparison with TRUS, mpMRI offers significantly better visualization of the prostate gland, but it is associated with higher costs, lower availability, and greater complexity of the procedure. Additionally, factors such as claustrophobia, medical implants, obesity, and inability to administer contrast due to allergies or kidney failure, can present further barriers to performing mpMRI.^14^ A cheaper, faster, and more accessible alternative to mpMRI, especially for prostate cancer screening, may be microUS,^15^;^16^ whose detection rate for PCa is comparable to that of mpMRI at all stages of advancement.^17^ Similarly to MRI, the standardized PRI-MUS protocol has been developed to unify the interpretation of images obtained with microUS.^11^ This protocol helps the examiner identify suspicious areas of the prostate indicative of PCa, standardizes the examination methodology, and includes a scoring system to stratify the risk of PCa. A limitation of the PRI-MUS protocol is that it primarily focuses on the peripheral zone (PZ) of the prostate. Therefore, cancer in other zones of the gland may be overlooked, although it should be noted that 70%–80% of PCas occur in the PZ.

Among the 439 patients included in the study, nearly half (44.6%) were suspected of having PCa based on microUS examination. In this group, 196 patients with a positive microUS result (44%) underwent mpMRI, which showed a suspicious lesion in 36% of cases, classified as PI-RADS 3 or higher. Nearly two-thirds of the patients (64%) had negative mpMRI results. In this group, 41% underwent biopsy, and over half of them had a positive result. Ultimately, 30% of the patients were diagnosed with csPCa, and nearly 70% with low-risk PCa. Therefore, a positive microUS result increased the likelihood of detecting not only non-csPCA but also csPCa. However, it should be noted that not all patients with positive microUS finding underwent mpMRI, so the results cannot be extrapolated to the entire population, and require verification in further studies. Among the patients with positive results on both mpMRI and microUS, nearly 30% showed discrepancies. An agreement is achieved when a lesion is visualized in both tests in the same location. The fact that 30% of the visualizations differed in location suggests some well-known limitations of conventional ultrasound, which also apply to the new technology of microUS. The US examination is operator-dependent and has a relatively low detection rate when a lesion is located outside the prostate PZ. After a thorough analysis, only 1 of the 9 discordant lesions was detected in the PZ according to mpMRI results. Overall, half of the patients in this group underwent prostate biopsy, which identified 1 case of csPCa, and 3 cases of low-risk PCa. It can be concluded that the discrepancy did not significantly affect the omission of csPCa, and both tests independently allowed for the detection of tumors at a very early stage of the disease. On the other hand, it can be assumed that a greater number of biopsy examinations among patients with suspected neoplastic changes on microUS would lead to a higher detection rate of csPCa.

Among the patients with a positive microUS result who did not undergo mpMRI, 10% were diagnosed with csPCa, and 39% had low-risk PCa. In the cases with a negative microUS result, the concordance with mpMRI was 79%. However, when the mpMRI result was positive, 40% of the cases showed discrepancies with microUS findings. Among the men with a positive mpMRI result, only 8% underwent prostate biopsy, and 1 case of csPCa was detected, which limits the ability to draw general conclusions. However, we may hypothesize that a negative microUS result carries a low risk of PCa detection, even when the mpMRI result is positive. In the entire group of patients with a negative microUS result, nearly two-thirds (64%) did not undergo mpMRI. However, after performing random biopsies, PCa was detected in 3 patients (low-risk only). Looking at the entire cohort of patients with a negative microUS result, it can be observed that among those who underwent biopsy, only 4.5% were diagnosed with PCa, and only 1% had csPCa. These findings are important, as they show that patients with a negative microUS result may not need to undergo immediate invasive prostate biopsy, thus avoiding all associated risks. Additionally, among the patients who underwent biopsy, csPCa was detected in only 1 case, which confirms previous research that demonstrated high negative predictive value of microUS.

In the group of 55 patients with a negative mpMRI result, 7% were diagnosed with csPCa, and 16% with non-csPCa. This suggests that a negative mpMRI result may require additional testing in some individuals. In the population with a negative microUS result, only 4.5% of the patients were diagnosed with PCa after biopsy, with only 1.2% being diagnosed with csPCa, and 3.2% with non-csPCa. The study also showed that with a negative mpMRI result, there is a high concordance with microUS. In light of these results, it can be concluded that 91% of the individuals with negative microUS results did not require further invasive diagnostic procedures. Our study also indicates that the PRI-MUS score of 4 or 5 significantly influences the decision to perform prostate biopsy, as such a score is associated with a high risk of PCa. We found that in the population of patients with a positive microUS result, low-risk PCa was detected in 42%, and csPCa in 14% of the participants. In the group of patients with a negative microUS result, only 4.5% had PCa detected, with csPCa diagnosed in just 1.2%, and non-csPCa in 3.2% of the cases. However, it should be noted that only a portion of the population with a negative microUS result underwent prostate biopsy. It seems that a negative result is associated with a low risk of missing csPCa.

The first multicenter prospective study was conducted by ^18^ and it compared the diagnostic effectiveness of microUS and mpMRI in a group of 1040 patients. Biopsies were performed for lesions rated as 3 or higher as per the PRI-MUS score for microUS and PI-RADS for mpMRI. The authors demonstrated that targeted biopsy under microUS guidance had higher sensitivity (94% vs 90%; *P* = 0.03) and higher negative predictive value (85% vs 77%; *P* = 0.04) than biopsy under mpMRI guidance. However, both techniques showed similar specificity (22% vs 22%; *P* = 0.45) and positive predictive value (44% vs 43%; *P* = 0.32) in detecting csPCa. Our study also showed a high agreement between negative results from microUS and mpMRI. Furthermore, in the group of patients with a negative microUS result, csPCa was detected in only 1 individual. This suggests that the patients who underwent microUS and received negative results do not need to undergo immediate invasive prostate biopsy.

An interesting study was published by ^19^ in which 159 patients who had undergone mpMRI and were suspected of having PCa were examined with microUS. Lesions identified on microUS were documented with the PRI-MUS protocol and compared with the mpMRI findings. Biopsy was performed independently of the lesions visualized on mpMRI. PCa was diagnosed in 71% of the patients, with csPCa (International Society of Urological Pathology score ≥2) found in 49% of the cases. According to these results, targeted biopsies under microUS guidance identified by 26% more aggressive lesions than nontargeted biopsies and by 16% more than nontargeted and MRI-targeted biopsies. In 17% of the patients, the targeted biopsy under MRI guidance gave a negative result, while the biopsy under microUS guidance detected cancer, with nearly 14% being csPCa. In a prospective, single-center study by ^20^ the accuracy of microUS in diagnosing PCa was evaluated in a group of 320 patients who had previously undergone mpMRI, where at least 1 lesion was rated as PI-RADS 3 or higher. csPCa was diagnosed in 36% of the patients. The sensitivity and negative predictive value of microUS were 89.7% and 81.5%, respectively, while the specificity and positive predictive value were 26% and 41%, respectively. A combination of targeted biopsy under microUS guidance and randomized biopsies would allow for the diagnosis of the same proportion of csPCa as a combination of targeted biopsies under MRI guidance and randomized biopsies (97.4%). In both groups, 3 cases of csPCa were diagnosed. ^21^ conducted the first randomized multicenter study which included 203 patients. It demonstrated that microUS was noninferior to mpMRI in detecting csPCa. In total, csPCa was diagnosed in 39% of the patients. Biopsies under microUS and MRI guidance detected csPCa in 73% and 76% of the individuals, respectively. With MRI-guided targeted biopsies, 9% of the PCa cases not found on microUS were detected. The microUS technique was as efficient as mpMRI, identifying 97% of the csPCa cases found with MRI-guided targeted biopsy.

### Study limitations

The study has some limitations that need to be accounted for. First, the patient population included was not homogeneous. All patients who underwent microUS imaging were included. On one hand, this significantly increased the sample size, but on the other, it also increased variability of the entire cohort, thus reducing the precision of the study. Another limitation is the fact that mpMRI was performed selectively and not on all patients included in the study, which could have affected the results regarding the agreement between the 2 methods. Third, the definition of csPCa used is not universal. In the study, lesions with the GS above 7 were classified as csPCa, basing the definition solely on the histological appearance of the tumor, which may conflict with definitions used by other authors.

Another limitation, universal to other US-based technologies, is the impact of the examiner’s experience on the obtained results and their accuracy. To minimize the effect of this factor, all microUS examinations were performed by 2 urologists with extensive experience in microUS and a very good understanding of the PRI-MUS protocol. Ultimately, there were phases of the study that were strongly influenced by the COVID-19 pandemic. Government-imposed limited access to standard care resulted in a reduced number of patients recruited, as well as fewer mpMRI examinations and prostate biopsies performed.

## CONCLUSIONS

The results of the study suggest that microUS is a promising and useful technology in the field of urological diagnostics. It shows the potential to become a cost-effective imaging method with a very high resolution. A positive result from microUS, even when MRI is negative, may indicate the need for prostate biopsy. On the other hand, our study suggests that a negative result from microUS indicates a low likelihood of csPCa. Obviously, based on the presented results, it cannot be concluded that there is no necessity for prostate biopsy associated with a negative microUS result. Further studies are necessary to evaluate the relationship between a negative microUS result and the risk of csPCa diagnosis.

## References

[BIBR-1] Sung H., Ferlay J., Siegel R.L. (2021). Global cancer statistics 2020: GLOBOCAN estimates of incidence and mortality worldwide for 36 cancers in 185 countries. CA Cancer J Clin.

[BIBR-2] Sekhoacha M., Riet K., Motloung P. (2022). Prostate cancer review: genetics, diagnosis, treatment options, and alternative approaches. Molecules.

[BIBR-3] Purysko A.S., Tempany C., Macura K.J. (2023). American College of Radiology initiatives on prostate magnetic resonance imaging quality. Eur J Radiol.

[BIBR-4] Rohrbach D., Wodlinger B., Wen J. (2018). High-frequency quantitative ultrasound for imaging prostate cancer using a novel micro-ultrasound scanner. Ultrasound Med Biol.

[BIBR-5] Postema A., Mischi M., Rosebe J., Wijkstra H. (2015). Multiparametric ultrasound in the detection of prostate cancer: a systematic review. World J Urol.

[BIBR-6] Fusco F., Emberton M., Arcaniolo D. (2023). Prostatic high-resolution micro-ultrasound: an attractive step-forward in the management of prostate cancer patients. Prostate Cancer Prostatic Dis.

[BIBR-7] Pavlovich C.P., Cornish T.C., Mullins J.K. (2014). High-resolution transrectal ultrasound: pilot study of a novel technique for imaging clinically localized prostate cancer. Urol Oncol.

[BIBR-8] Zhang M., Wang R., Wu Y. (2019). Micro-ultrasound imaging for accuracy of diagnosis in clinically significant prostate cancer: a meta-analysis. Front Oncol.

[BIBR-9] Abouassaly R., Klein E.A., El-Shefai A., Stephenson A. (2020). Impact of using 29 MHz high-resolution micro-ultrasound in real-time targeting of transrectal prostate biopsies: initial experience. World J Urol.

[BIBR-10] A Basso Dias, S Ghai (2023). Micro-ultrasound: current role in prostate cancer diagnosis and future possibilities. Cancers (Basel.

[BIBR-11] Ghai S., Eure G., Fradet V. (2016). Assessing cancer risk on novel 29 MHz micro-ultrasound images of the prostate: creation of the micro-ultrasound protocol for prostate risk identification. J Urol.

[BIBR-12] Weinreb J.C., Barentsz J.O., Choyke P.L. (2016). PI-RADS prostate imaging-reporting and data system: 2015, version 2. Eur Urol.

[BIBR-13] Würnschimmel C., Chandrasekar T., Hahn L. (2023). MRI as a screening tool for prostate cancer: current evidence and future challenges. World J Urol.

[BIBR-14] Visschere PJL, A BriganA, JJ Füberer (2016). Role of multiparametric magnetic resonance imaging in early detection of prostate cancer. Insights Imaging.

[BIBR-15] Avolio P.P., Lughezzani G., Anidjar M. (2023). The diagnostic accuracy of micro-ultrasound for prostate cancer diagnosis: a review. World J Urol.

[BIBR-16] Maffei D., Fasulo V., Avolio P.P. (2023). Diagnostic performance of micro-ultrasound at MRI-guided confirmatory biopsy in patients under active surveillance for low-risk prostate cancer. Prostate.

[BIBR-17] Sountoulides P., Pyrgidis N., Polyzos S.A. (2021). Micro-ultrasound-guided vs multiparametric magnetic resonance imaging-targeted biopsy in the detection of prostate cancer: a systematic review and meta-analysis. J Urol.

[BIBR-18] Klotz L., Lughezzani G., Maffei D. (2021). Comparison of micro-ultrasound and multiparametric magnetic resonance imaging for prostate cancer: a multicenter, prospective analysis. Can Urol Assoc J.

[BIBR-19] Wiemer L., Hollenbach M., Heckmann R. (2021). Evolution of targeted prostate biopsy by adding micro-ultrasound to the magnetic resonance imaging pathway. Eur Urol Focus.

[BIBR-20] Lughezzani G., Maffei D., Saita A. (2021). Diagnostic accuracy of microultrasound in patients with a suspicion of prostate cancer at magnetic resonance imaging: a single-institutional prospective study. Eur Urol Focus.

[BIBR-21] Hofbauer S.L., Luger F., Harland N. (2022). A non-inferiority comparative analysis of micro-ultrasonography and MRI-targeted biopsy in men at risk of prostate cancer. BJU Int.

